# Chronic Oxidative Stress Increases Growth and Tumorigenic Potential of MCF-7 Breast Cancer Cells

**DOI:** 10.1371/journal.pone.0087371

**Published:** 2014-01-28

**Authors:** Prathap Kumar S. Mahalingaiah, Kamaleshwar P. Singh

**Affiliations:** Department of Environmental Toxicology, The Institute of Environmental and Human Health (TIEHH), Texas Tech University, Lubbock, Texas, United States of America; Wayne State University, United States of America

## Abstract

Accumulating evidence suggests that exposures to elevated levels of either endogenous estrogen or environmental estrogenic chemicals are associated with breast cancer development and progression. These natural or synthetic estrogens are known to produce reactive oxygen species (ROS) and increased ROS has been implicated in both cellular apoptosis and carcinogenesis. Though there are several studies on direct involvement of ROS in cellular apoptosis using short-term exposure model, there is no experimental evidence to directly implicate chronic exposure to ROS in increased growth and tumorigenicity of breast cancer cells. Therefore, the objective of this study was to evaluate the effects of chronic oxidative stress on growth, survival and tumorigenic potential of MCF-7 breast cancer cells. MCF-7 cells were exposed to exogenous hydrogen peroxide (H_2_O_2_) as a source of ROS at doses of 25 µM and 250 µM for acute (24 hours) and chronic period (3 months) and their effects on cell growth/survival and tumorigenic potential were evaluated. The results of cell count, MTT and cell cycle analysis showed that while acute exposure inhibits the growth of MCF-7 cells in a dose-dependent manner, the chronic exposure to H_2_O_2_-induced ROS leads to increased cell growth and survival of MCF-7 cells. This was further confirmed by gene expression analysis of cell cycle and cell survival related genes. Significant increase in number of soft agar colonies, up-regulation of pro-metastatic genes *VEGF, WNT1* and *CD44*, whereas down-regulation of anti-metastatic gene *E-Cadherin* in H_2_O_2_ treated MCF-7 cells observed in this study further suggests that persistent exposure to oxidative stress increases tumorigenic and metastatic potential of MCF-7 cells. Since many chemotherapeutic drugs are known to induce their cytotoxicity by increasing ROS levels, the results of this study are also highly significant in understanding the mechanism for adaptation to ROS-induced toxicity leading to acquired chemotherapeutic resistance in breast cancer cells.

## Introduction

Breast cancer is the most commonly diagnosed cancer in women worldwide and also the leading cause of mortality in US women [1]–[3]. Tremendous progress have been made over the last decades in understanding the biology of breast cancer, however the mechanism for growth and progression of breast cancer with acquisition of invasive and metastatic phenotypes and therapeutic resistance are still not fully understood.

Evidence suggests that multiple intrinsic and extrinsic risk factors and their interactions are involved in breast cancer development and progression [4], [5]. Intrinsic factors including all known genetic susceptibility variants account for 20–25% breast cancer incidence [6]. Long-term exposure to extrinsic or environmental factors has been attributed for more than 70% of sporadic breast cancers [7].

The accumulating evidence suggest a potential link between environmental chemicals and breast cancer risk [1]. Majority of environmental chemicals mimics estrogenic activity and therefore classified as xenoestrogens. Some of the well-established xenoestrogens such as Diethylstilbesterol [8], Polychlorinated biphenyls [1], [9], Bisphenol [8], Organochlorine pesticides [9], have been linked with breast cancer. Because of the lipophilic nature, these xenoestrogens tends to bio-accumulate and persist in the body for longer time and therefore increases the potential risk for breast cancer development [10].

While the role of both elevated levels of endogenous estrogen and exposure to xenoestrogens in breast cancer development is well known, the mechanism of their carcinogenic effect is poorly understood. Various mechanisms have been proposed for estrogen-induced growth and development of breast cancer. For example estrogen has been shown to increase cell proliferation of both normal breast epithelial cells and breast cancer cells [11]–[14]. Estrogen has been shown to activate mitogenic signaling [11], [15], activation of oncogenes [16]–[18], inactivation of tumor suppressor genes [15], [16], [19], chromosomal aberrations (both structural and numerical) [15], and alterations in epigenetic markers [14]. Both estrogen receptor-dependent and independent pathways have been proposed for these biological responses of estrogens [15]. Receptor-dependent carcinogenic action of estrogen involves estrogen receptor-mediated aberrant regulation of estrogen responsive genes leading to aberrant expression of cell proliferation and DNA repair genes, that consequently leads to increased cell proliferation and accumulation of DNA damage ultimately causing cell transformation [20]. Receptor-independent pathway involves cytochrome P450 mediated oxidative metabolism of estrogens resulting in generation of genotoxic metabolites and reactive oxygen species [15], [21]. These metabolites by themselves after forming DNA adducts or ROS generated during estrogen metabolism as a signalling molecules also leads to increased cell proliferation and DNA damage and consequently cell transformation [22], [23]. Increased lipid peroxidation and up-regulation of antioxidant enzymes prior to mammary tumor development in ACI rat model of estrogen-induced mammary cancer also support potential role of oxidative stress in breast cancer [24]. Detection of significantly higher levels of environmental estrogenic chemicals and 8–hydroxy, 2-deoxy guanosine, a classical indicator for oxidative DNA damage in human breast cancer samples when compared to normal cells from same patient further strengthens the potential role of xenoestrogens-induced ROS and ROS-induced DNA damage in breast cancer development and/or progression [25], [26]

In addition to estrogen and xenoestrogens- mediated ROS increase, endogenous factors like mitochondrial dysfunction in cancer cells [27], inadequate blood supply due to lack of proper vascular network [28] and therapeutic intervention [29] may also increase ROS levels leading to oxidative stress in breast cancer cells. Phagocytic cells including macrophages and poly morpho nuclear leucocytes (PMNs) are known to generate ROS for their phagocytic activity and significant increase in their numbers has been reported in breast tumors compared to normal breast tissue [30]. Hence, estrogens and estrogen-like chemicals-mediated activation of phagocytic cells also acts as an additional source of increased ROS exposure for breast cancer cells [18]. Use of cancer chemotherapeutic drugs may also increase oxidative stress burden for breast cancer cells [31]. Recent reports suggest involvement of ROS in both intrinsic and/or acquired resistance to chemotherapeutic agents in breast cancer cells [32].

These reports suggest that oxidative stress due to increased ROS by estrogens or estrogenic chemicals and other endogenous or exogenous factors may play a role in breast cancer development, progression and resistance to some chemotherapeutic drugs. Since both endogenous and exogenous factors may act by multiple mechanisms, there is very little evidence on the direct involvement of ROS in breast cancer growth and progression. Most of the previous studies used short-term models to study the response of breast cancer cells to oxidative stress [29], [33], [34]. However, the effects of long-term/persistent exposure to oxidative stress due to increased ROS on breast cancer growth, tumorigenicity and metastatic potential is not known.

Therefore the objective of this study was to evaluate the effect of exogenous hydrogen peroxide (H_2_O_2_)-induced acute and chronic oxidative stress on the growth, survival and tumorigenicity of MCF-7 breast cancer cells.

## Materials and Methods

### Chemicals

Hydrogen peroxide, 3-(4,5-dimethylthiazol-2-yl)-2,5-diphenyltetrazolium bromide (MTT), and 2′,7′dichlorodihydrofluorescein diacetate (DCFH-DA) were purchased from Sigma (St. Louis, MO). Dulbecco's modified Eagle's medium (DMEM), trypsin/EDTA, Fetal Bovine Serum (FBS), antibiotic/antimycotic solution, and Trizol reagent were purchased from Invitrogen Inc. (Carlsbad, CA). PCR reagents were purchased from BioRad, Inc. and cell cycle reagent was obtained from Millipore (Hayward, CA).

### Cell culture and treatments

MCF-7 cells were purchased from ATCC and were maintained in phenol red free DMEM/F-12 medium supplemented with 10% FBS and 1% antibiotic and antimycotic solution. Cultures were grown and maintained at 37°C in a humidified atmosphere containing 5% CO_2_. Actively growing MCF-7 cells were seeded in 25 cm^2^ flasks. Cells grown up to 40–60% confluence were treated with 25 µM and 250 µM concentrations of H_2_O_2_ in fresh culture media. H_2_O_2_ concentration of 25 µM as low dose and 250 µM as high dose were selected from a range of doses tested to include non-cytotoxic and significantly cytotoxic doses respectively.

For acute exposure, MCF-7 cells were exposed to H_2_O_2_ for 24 hours and then used for analysis. For chronic exposure, H_2_O_2_ treated MCF-7 cells were sub-cultured after every 6 days when the cells grown to 70–80% confluence and the process was repeated for 3 months. Both acute and chronic treatment cultures were maintained and treated in triplicate. Parallel cultures were grown and maintained as a passage matched controls.

### Measurement of reactive oxygen species

ROS production was measured using 2′,7′–dichlorofluorescein diacetate (DCFH-DA) method. DCFH-DA is a stable, fluorogenic and non-polar compound which can readily diffuse into the cells and get deacetylated by intracellular esterases to a non-fluorescent 2′,7′-dichlorodihydrofluorescein (DCFH) which is later oxidized by intracellular ROS into highly fluorescent 2′,7′-dichlorofluorescein (DCF). The intensity of fluorescence is proportional to intracellular ROS levels. MCF-7 cells were seeded at a density of 10^4^ cells per well in 96 well plates and were allowed to attach overnight. Next day cells were washed with 1X PBS and incubated with DCFH-DA in a final concentration of 10 µM for 30 minutes. Cells were rinsed with PBS and then treated with H_2_O_2_ solution in 1% FBS supplemented PBS for 30 minutes. DCF fluorescence intensity was measured using Biotek Synergy-4 microplate reader at excitation wavelength of 485 nm and emission wavelength of 535 nm

### Cell count and cell viability assay

Cell count analysis and cell viability by MTT assay were performed to assess the effect of H_2_O_2_ exposure on cell proliferation and growth. For acute exposure study, MCF-7 cells at a density of 200,000 cells per well were seeded in 6 well plate in triplicate and allowed to attach and grow for 2 days to reach 60-70% confluency. Treatment group of cells were treated with H_2_O_2_ at concentration of 25 µM and 250 µM. After 24 hours of treatment, cells were trypsinised and suspended in cell culture media and cell count was performed using Cellometer Auto T4 cell counter (Nexcelom Biosciences). For chronic exposure study, MCF-7 cells treated with H_2_O_2_ for 3 months were used for seeding in 6 well plates and then similar cell counting process was repeated. Both acute and chronic experiment was repeated twice.

Cell viability assay was performed using 3-(4,5-dimethylthiazol-2-yl)-2,5-diphenyltetrazolium bromide (MTT) dye reduction method to determine cytotoxicity of H_2_O_2_. MCF-7 cells (2000 cells) were seeded in 96 well flat bottom culture plates and allowed to attach and grow for 24 hours at 37°C with 5% CO_2_ in a cell culture incubator. Cells were treated with H_2_O_2_ at concentrations of 25 µM and 250 µM. After 24 hours of treatment, 20 µL of MTT solution (10 mg/mL) was added to each well and plate was incubated for 3 hours at 37°C with 5% CO_2_ level. To dissolve insoluble formazan crystals formed in mitochondria, media was removed completely, and 150 µL of DMSO was added to each well and then plate was incubated with gentle shaking for 5 minutes. The resulting color intensity was measured at 570 and 630 nm absorbance using Biotek Synergy-4 microplate reader. Each treatment was performed in triplicates and experiment was repeated at least twice.

### Cell cycle analysis by flow cytometry

To determine the effect of acute exposure to H_2_O_2_ on the cell cycle by flow cytometry, 500,000 MCF-7 cells were seeded in 25 cm^2^ culture flasks and treated with 25 µM and 250 µM concentrations of H_2_O_2_ for 24 hours. Similarly equal number of cells chronically exposed to H_2_O_2_ and their passage matched untreated control were seeded in 25 cm^2^ flask. Semi-confluent actively growing cells with acute and chronic exposure to H_2_O_2_ were collected and fixed in 70% ethanol for at least 24 hours at 4°C. Just before flow cytometry analysis, fixed cells were collected by centrifugation, washed with ice cold 1X PBS followed by staining with Guava cell cycle reagent (Millipore) and analysed in Guava Easy-Cyte HT Flow Cytometer (Millipore). Cell cycle analysis was carried out by counting 5000 events and acquired data was analysed using Guava Incyte software (Millipore). All samples were analysed in triplicates and experiment was repeated twice.

### RNA extraction and quantitative real-time PCR (q RT-PCR)

Actively growing cells of treatment and control groups were used to isolate total RNA by Trizol method and quantified by Nanodrop 1000 spectrophotometer (Thermo Scientific). Single step qRT-PCR amplifications were performed in an iCycler IQ (Bio-Rad) real time PCR machine using one-step RT-PCR kit with SYBR green and 200 ng total RNA following the manufacturer's protocol (Bio-Rad). Real-time PCR machine was programmed for reverse transcription at 50°C for 15 min, denaturation and RT enzyme inactivation at 95°C for 5 min, followed by 40 cycles each containing 10 seconds for denaturation at 95°C and 30 seconds for annealing and extension at 60°C. Specificity of the PCR products was confirmed by melt curve analysis. Data were normalized to GAPDH Ct values from the same sample and the fold-changes in gene expression were calculated by using the delta-delta Ct method [35]. A non-template control was included in each experiment. Primer sequences used for analysis of gene expression changes have been listed in Table 1.

**Table 1:** List of genes with their forward and reverse primer sequences used for the gene expression analysis by real- time quantitative PCR.

Gene	Forward primer sequence (5′-3′)	Reverse primer sequence (5′-3′)	Size (base pair)
*GAPDH*	GGTGGTCTCCTCTGACTTCAACA	GTTGCTGTAGCCAAATTCGTTGT	116
*CyclinD1*	AACTACCTGGACCGCTTCCT	CCACTTGAGCTTGTTCACCA	204
*Survivin*	AGCCAGATGACGACCCCATT	GCAACCGGCCGAATGCTTTT	119
*PARP1*	GCCCTAAAGGCTCAGAACGA	AAGGCACTTGCTGCTTGTTG	112
*Bcl2*	GGATGCCTTTGTGGAACTG	AGCCTGCAGCTTTGTTTCAT	231
*CD44*	CCCAGATGGAGAAAGCTCTG	GTTGTTTGCTGCACAGATGG	113
*CDH1*	TGAGTGTCCCCCGGTATCTT	GAATCATAAGGCGGGGCTGT	112
*WNT1*	CCTCCACGAACCTGCTTACA	TCCCCGGATTTTGGCGTATC	108
*VEGF*	AGTTCGAGGAAAGGGAAAGGG	GGAGGCTCCAGGGCATTAGA	100

### Soft agar assay for colony formation

Colony formation assay on soft agar was performed to determine the effect of chronic exposure to H_2_O_2_ on anchorage independent growth of MCF-7 cells. To prepare the base layer, 1.5 ml of 0.5% agar in DMEM media was added to 6 well plates and allowed to polymerize. Top layer was prepared by making 0.35% agar in cell culture media, and was cooled to about 40°C and then 5000 cells were mixed in 1.5 mL of top layer agar and plated over the base layer. Plates were allowed to solidify and then incubated at 37°C. Colony formation and growth on soft agar was monitored daily by microscopic observation. Colonies were counted and images were taken at day 14 when colonies of cells reached in size to the level that they were clearly visible under microscope. The numbers of colonies in treatment groups were converted to percentage of control (by considering control as 100%).

### Statistical analysis

To determine whether the differences observed were statistically significant, a t-test (two-tailed, paired samples for means, and hypothesized difference of 0) was performed on the data. An ANOVA was performed to determine if the source of variation in the data was between or within treatment groups. Alpha (α) was set at 0.05 for all statistical tests and data with p<0.05 were considered as significantly different.

## Results

### Confirmation of H_2_O_2_- induced intracellular ROS

**Figure 1:**
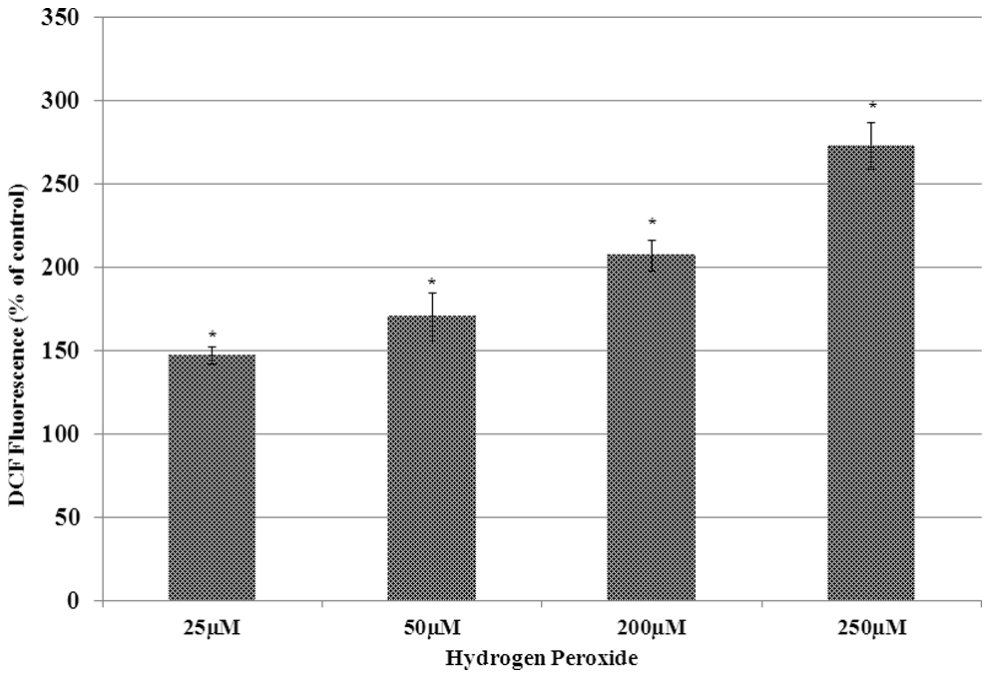
Bar graph representation of data from DCFH-DA assay on H_2_O_2_ treated MCF-7 cells. Cell treatment and DCFH-DA assay was performed as described in materials and methods. Statistically significant (p<0.05) changes are indicated by symbol *.

To evaluate and confirm exogenous H_2_O_2_ induced intracellular ROS, MCF-7 cells were seeded in 96 well plates and pre-incubated with redox sensitive and fluorogenic dye (DCFH-DA). H_2_O_2_ treatment resulted in significant and dose-dependent increase in DCF fluorescence (Figure 1).

### Effect of H_2_O_2_ on cell growth

**Figure 2:**
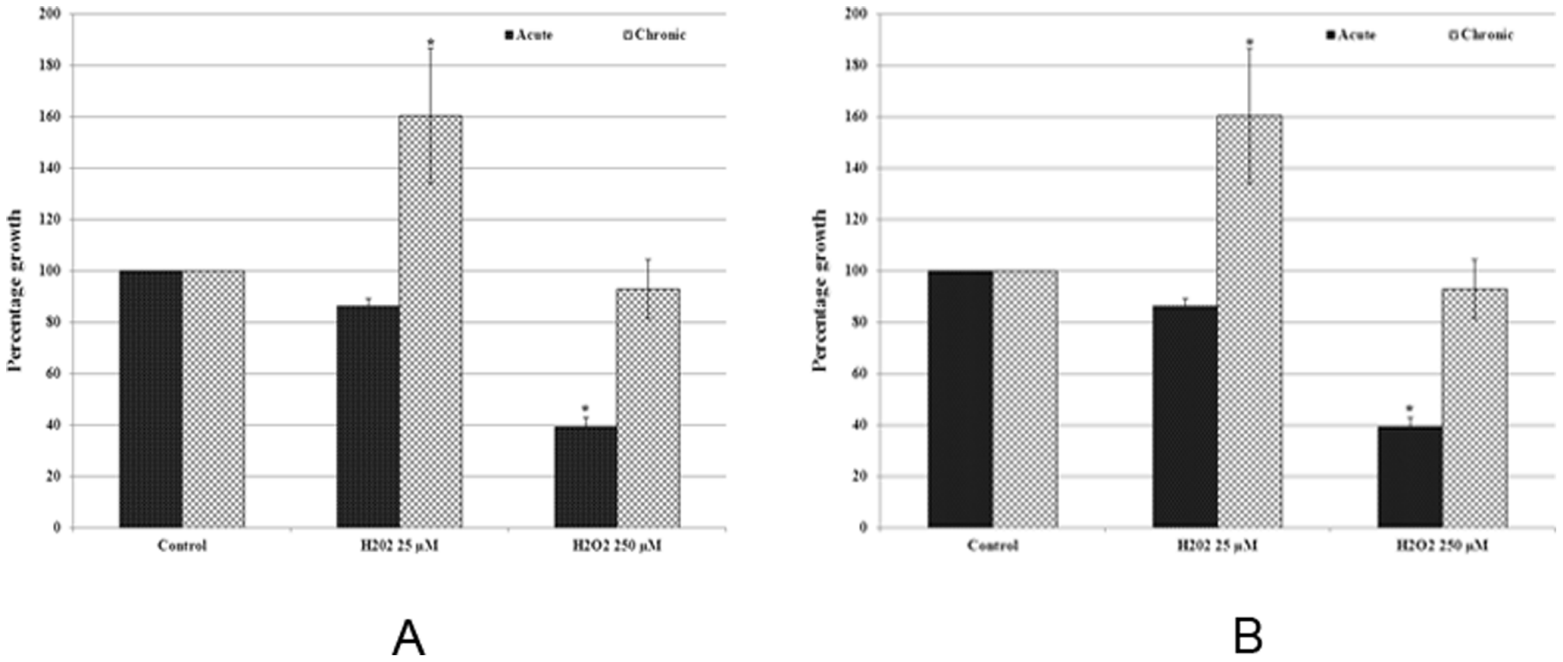
Bar graph representation of cell growth data from cell count analysis (Figure 2A), and MTT assay (Figure 2B) of MCF-7 cells with acute and chronic exposure to H_2_O_2_. Values for cell count and MTT assay were converted into percentage of control (control = 100%). The error bars represent the standard error of the mean (±SEM). Statistically significant (p<0.05) changes are indicated by symbol *.

The effect of acute and chronic exposure to H_2_O_2_ on the growth of MCF-7 cells was analysed by cell count and MTT assay and results are presented in Figure 2A and 2B. The cell count data revealed that, the lower dose of H_2_O_2_ resulted in a small statistically insignificant decrease in growth of cells with acute exposure to H_2_O_2_ for 24 hours. The same dose of H_2_O_2_ after chronic exposure for 3 months resulted in 60.11% increase in cell growth (Figure 2A).The exposure to higher dose of H_2_O_2_ (250 µM) resulted in statistically significant (p<0.05) decrease in growth of cells by 60.86% after acute exposure, however chronic exposure (3 months) caused 92.85% growth of cells, suggesting thereby the increased adaptability of cells due to chronic exposure (Figure 2B).

In order to further confirm cell count data, the MTT assay was performed. The results of MTT assay revealed decreased cell viability by 20.86% and 63.78% in cells with acute exposure to 25 and 250 µM H_2_O_2_ respectively. This decrease in cell viability by H_2_O_2_ exposure was statistically significant in higher doses. In contrast to a significant decrease in cell viability as observed in acute exposure, the chronic exposure to lower doses of H_2_O_2_ resulted in statistically significant (p<0.05) increase by 34.65% in viability of cells, and higher dose did not induce any significant decrease (19.20%) in cell viability (Figure 2B). Therefore the results of MTT assay further confirmed the H_2_O_2_ induced toxicity in acute exposure and increased cell adaptability to H_2_O_2_ in chronic exposure, as revealed by cell count data.

### Effect of H_2_O_2_ on cell cycle of MCF-7 cells

**Figure 3:**
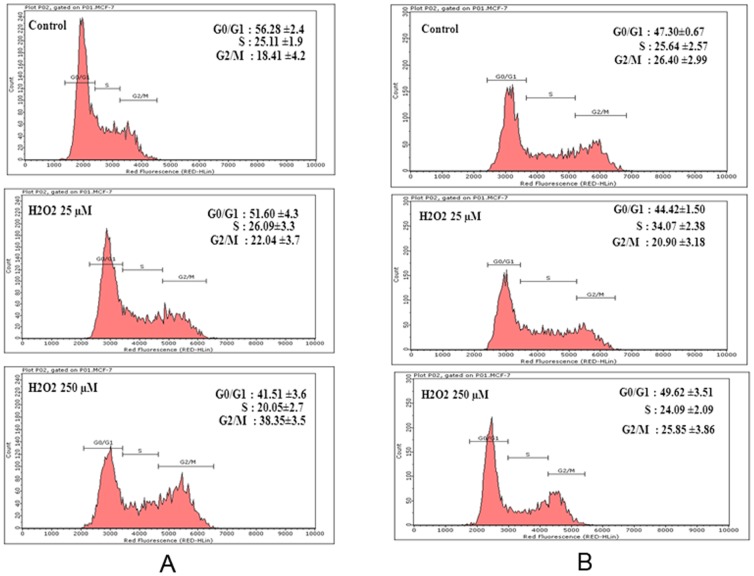
Flow cytometry histograms representing MCF-7 cells population in G0/G1, S, and G2/M phases of cell cycle after acute (Figure 3A) and chronic (Figure 3B) exposure to H_2_O_2_. Cells treated with H_2_O_2_ or untreated control were collected, fixed, and stained for cell cycle analysis by flow cytometry as described in Materials and Methods Section. Percentage of cells in G0/G1, S and G2/M phase of cell cycle from each histogram represent the average value from three independent experiments.

Cell cycle analysis by flow cytometry was performed to further understand the effect of acute and chronic exposure to H_2_O_2_ on proliferation of MCF-7 cells. The percentage of S phase cells in control group was 25%, whereas acute exposure to lower (25 µM) and higher (250 µM) doses of H_2_O_2_ resulted in 26% and 19.05% of cells in S phase, respectively (Figure 3A). Similarly, as compared to 18.41% G2/M phase cells in control, there was a dose-dependent increase with 22.04% and 38.35% G2/M cells in 25 µM and 250 µM H_2_O_2_ treatment groups respectively (Figure 3A). The observed decrease in S Phase and increase in G2/M phase cells by acute exposure to 250 µM H_2_O_2_ as compared to control was statistically significant (p<0.05).

As compared to control with 25.64% of cells in S phase, the chronic exposure to lower dose of H_2_O_2_ resulted in 36.08% cells in S phase (Figure 3B). This suggests that there was a 40.71% increase in S phase cells due to chronic exposure to low dose of H_2_O_2_, and this increase was statistically significant. The percentage of G2/M cells in control was 26.41%, whereas cells exposed to low dose of H_2_O_2_ has 20.90% cells in G2/M phase, suggesting thereby a decrease of 20.86% in G2/M phase cells due to H_2_0_2_ treatment. In contrast to acute treatment that led to statistically significant (p<0.05) decrease in S phase and increase in G2/M phase, the chronic exposure to higher dose of H_2_O_2_ resulted in no significant change in percentage of cells in S and G2/M phase as compared to control (Figure 3B). This further suggests the increased adaptability of cells due to chronic exposure.

### Effect of H_2_O_2_ on expression of genes

**Figure 4:**
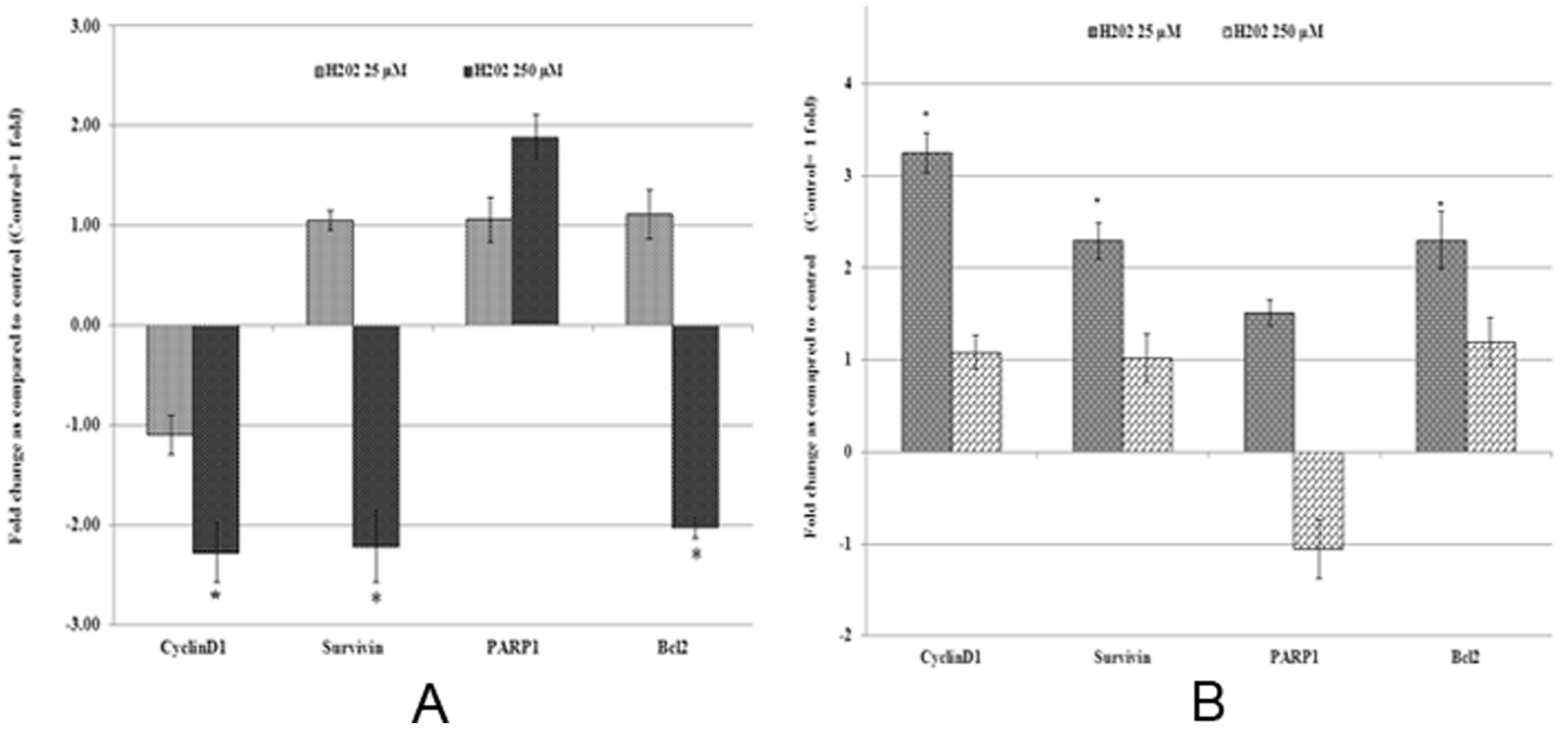
Real time quantitative reverse transcription PCR analysis of gene expression of cell growth and survival related genes. Total RNA isolated from MCF-7 cells with acute and chronic exposure to H_2_O_2_ was used to perform one step real-time quantitative reverse transcription PCR as described in materials and methods. Cycle threshold value (Ct value) of each gene was normalized to the Ct value of housekeeping gene GAPDH obtained from the same sample. The gene expression in fold change was calculated and histogram was plotted using the means of triplicate values. Results of acute (Figure 4A) and chronic (Figure 4B) exposure were presented in separate histograms. Statistically significant change (p<0.05) in gene expression in treated groups as compared to the untreated control is indicated by symbol *.

**Figure 5:**
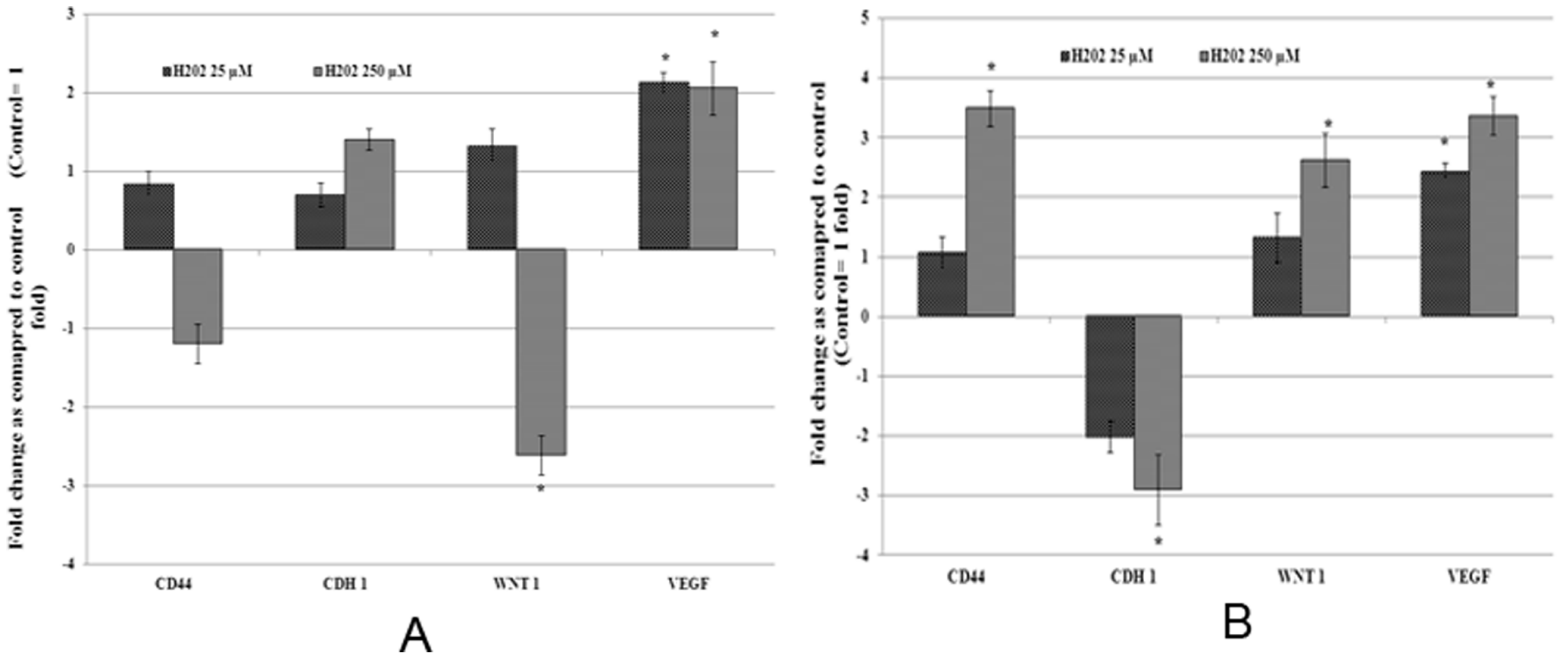
Real time quantitative reverse transcription PCR analysis of gene expression of metastasis related genes. Total RNA isolated from MCF-7 cells with acute and chronic exposure to H_2_O_2_ was used to perform one step real-time quantitative reverse transcription PCR as described in materials and methods. Cycle threshold value (Ct value) of each gene was normalized to the Ct value of housekeeping gene GAPDH obtained from the same sample. The gene expression in fold change was calculated and histogram was plotted using the means of triplicate values. Results of acute (Figure 5A) and chronic (Figure 5B) exposure were presented in separate histograms. Statistically significant change (p<0.05) in gene expression in treated groups as compared to the untreated control is indicated by symbol *.

To further understand the molecular basis of H_2_O_2_ induced toxicity in acute exposure and increased adaptability of cells due to chronic exposure, the expression of genes related to cell growth, cell survival, and metastasis was measured at transcript level. A summary of gene expression changes is given in Figures 4 and 5. Details of gene expression changes induced by acute and chronic exposure to H_2_O_2_ are categorised under the following sub-headings.

### Cell growth and survival related genes

*CyclinD1*, *Survivin, Poly (ADP-ribose) polymerase 1(PARP1)* and *Bcl2* were used in this study as representative for cell cycle and survival related genes. The acute exposure to lower dose of H_2_O_2_ did not cause any significant change in expression of any of these genes analysed. However, the higher dose of H_2_O_2_ resulted in statistically significant (p<0.05) decrease in the expression of *CyclinD1* by −2.28, *Survivin* by −2.22 and *Bcl2* by −2.02 folds (Figure 4A). Statistically insignificant increase by 1.88 fold in the expression of *PARP1* was observed in cells acutely exposed to 250 µM H_2_O_2_. In contrast to acute exposure, the chronic exposure to low dose of H_2_O_2_ resulted in a statistically significant (p<0.05) increase in the expression of *CyclinD1* by 3.25, *Survivin* by 2.29 and *Bcl2* by 2.3 folds (Figure 4B). In higher dose of H_2_O_2_, expression levels of all these genes were comparable to untreated control. The gene expression data showing no change in acute exposure but increased expression in chronic exposure with low dose H_2_O_2_, and decreased expression of these genes in acute exposure whereas no change in chronic exposure further confirms at the molecular level the increased cell adaptability and/or growth due to chronic exposure to H_2_O_2_.

### Metastasis related genes

The expression of metastasis marker genes *CD44, CDH1 (E-Cadherin), WNT1* and *Vascular endothelial growth factor (VEGF)* were analysed to evaluate the effects of H_2_O_2_ induced oxidative stress on metastatic potential of MCF-7 cells. Among these genes the expression of *VEGF* was significantly increased by 2.12 fold and 2.06 fold in cells with acute exposure to low and high dose of H_2_O_2_ respectively (Figure 5A), whereas the expression of WNT1 was significantly down-regulated in cells with acute exposure to high dose of H_2_O_2_. In contrast to acute exposure, the chronic exposure to high dose of H_2_O_2_ resulted in statistically significant increase in the expression of pro-metastatic genes *CD44* by 3.49 folds, *WNT1* by 2.62 folds and *VEGF* by 3.36 folds (Figure 5B). The expression of anti-metastatic gene *CDH1* was significantly down regulated by −2.9 fold as compared to control. The gene expression data suggest that chronic exposure to H_2_O_2_ not only increases growth and survival, but also increases metastatic potential of MCF-7 breast cancer cells.

### Effect of H_2_O_2_ on tumorigenicity of MCF-7 cells evaluated by colony formation on soft agar

**Figure 6:**
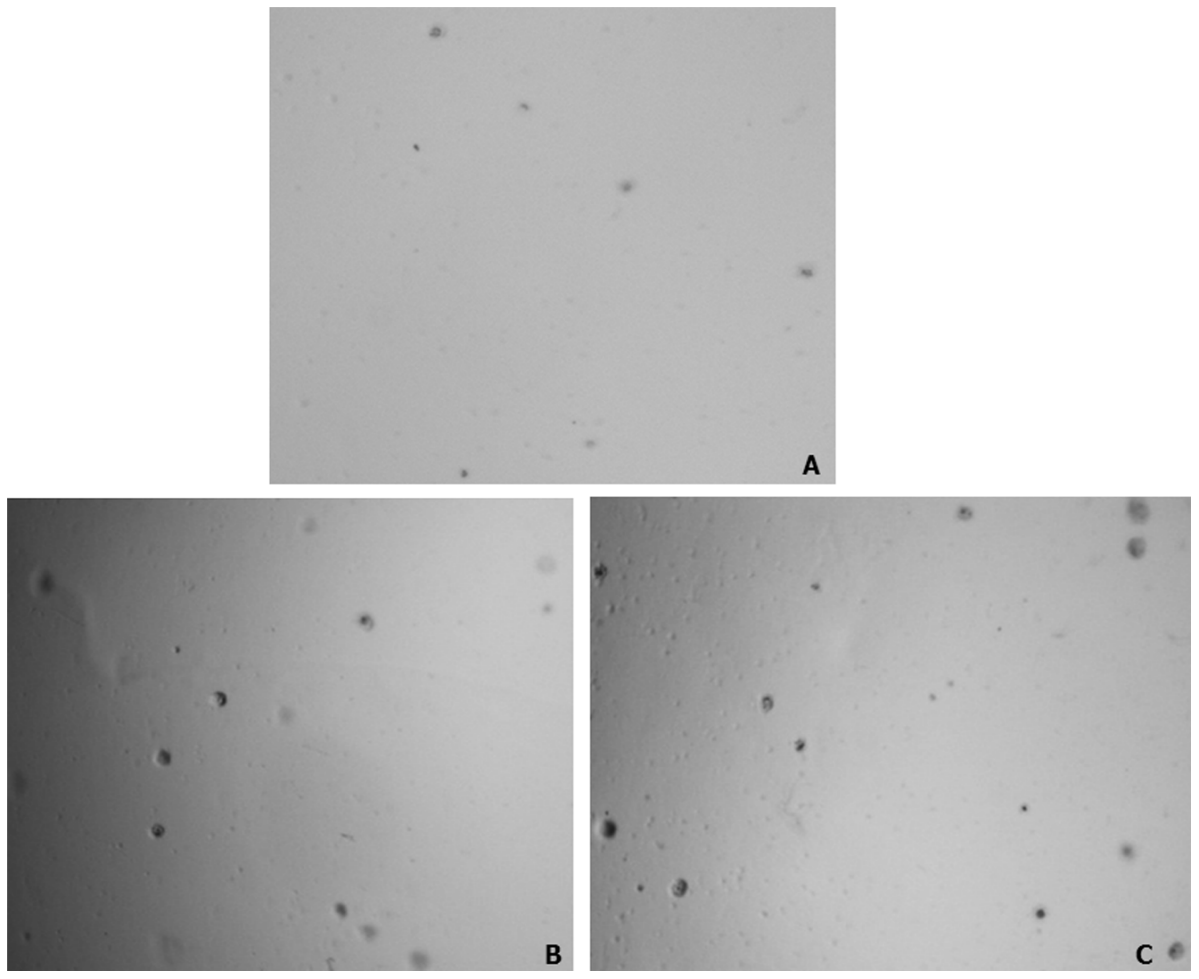
Representative photographs (40X magnification) of soft agar grown colonies. Actively growing MCF-7 cells with chronic exposure to H_2_O_2_ were harvested, plated, and grown in soft agar as described in materials and methods section. The representative images of colonies from control, cells chronically treated with 25 µM and 250 µM of H_2_O_2_ are given in panel A, B, and C respectively.

**Figure 7:**
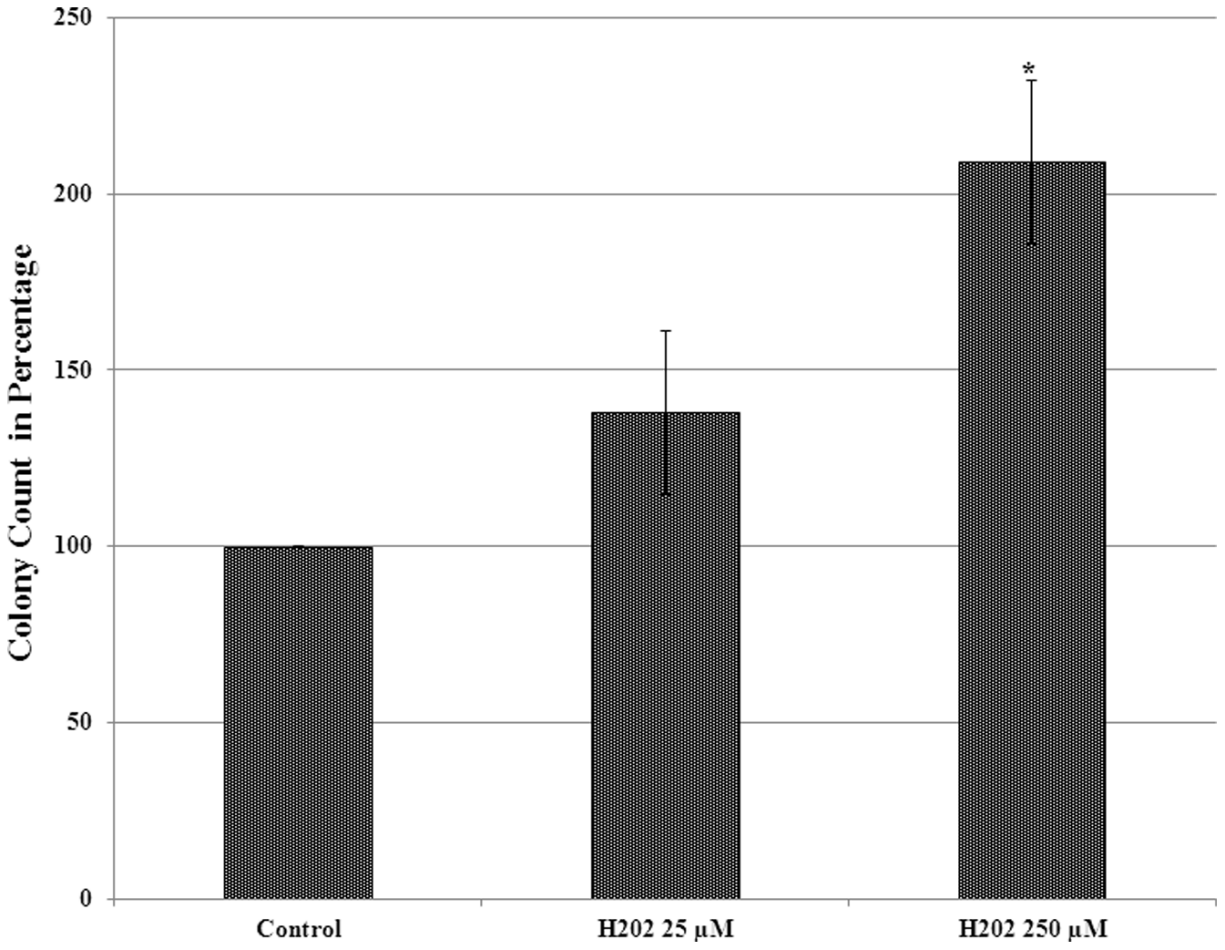
Histogram showing the effect of chronic exposure to H_2_O_2_ in MCF-7 on colony counts in soft agar assay. Colony numbers were converted into percentage of control (control = 100%). The error bars represent the standard error of the mean (±SEM). Statistically significant changes (p<0.05) when compared to untreated control are indicated by symbol *.

Soft agar assay was performed to determine the effect of chronic exposure to H_2_O_2_ induced oxidative stress on tumorigenic phenotype of MCF-7 cells. The result of soft agar assay revealed a significant increase in number of soft agar grown colonies from cells exposed to both low as well as high dose of H_2_O_2_ as compared to untreated control MCF-7 cells. Representative images of colonies from each treatment and control are given in Figure 6 and the percentage of colony number increase is given as histogram in Figure 7. As compared to untreated control, a 38% and 109% increase in number of colonies was observed from cells chronically treated with low and high dose H_2_O_2_ respectively (Figure 7). In addition to the increase in number of colonies, the size of soft agar colonies from H_2_O_2_ treated cells were also appear to be increased as compared to control (Figure 6). However, there was no distinct difference in the size of soft agar grown colonies between cells treated with low and high doses of H_2_O_2_. The result of soft agar assay suggests that chronic exposure to H_2_O_2_ not only increases the growth but also the tumorigenicity of MCF-7 cells.

## Discussion

The most important and novel finding that emerged from this study is that the chronic exposure to oxidative stress leads not only to increased cell growth and survival, but also in increased tumorigenic potential of MCF-7 breast cancer cells. Since both endogenous and exogenous risk factors of breast cancer including estrogenic chemicals may act by multiple mechanisms, the contribution of increased ROS in these biological responses is difficult to quantify. Therefore in this study we exposed MCF-7 breast cancer cells directly to the H_2_O_2_ induced oxidative stress and our data suggest that increased ROS by itself significantly contribute in induction of growth, survival and tumorigenic potential of MCF-7 cells.

The response of breast cancer cells to ROS has been previously examined, but these studies focused on short-term treatment models [29], [33], [34]. To our knowledge this is the first report providing direct evidence for the role of chronic exposure to increased ROS in increasing cell growth, survival and tumorigenicity. Exogenous H_2_O_2_ administration is known to increase highly reactive hydroxyl radical and has been used extensively to induce oxidative stress in different in-vitro and animal models [36]–[38]. MCF-7 cells are estrogen responsive human breast adenocarcinoma cells and have been tested using H_2_O_2_ exposure and reported as a suitable in-vitro model of choice for analysis of oxidative stress inducing chemicals [37], [39]. Hence we selected H_2_O_2_ as an exogenous source to expose MCF-7 cells to increased levels of intracellular ROS for acute and chronic period.

ROS are known to elicit broad spectrum of cellular response depending on concentration and duration of exposure. Low concentrations of ROS can acts as a secondary messengers and stimulate survival and proliferation of multiple cell types [40]. In addition, ROS mediates mitogenic activity of cells through regulation of cytosolic calcium concentration, phosphorylation of proteins, and activation of transcription factors like NF-kβ and AP-1 [41], [42]. Similar mitogenic response of estrogen-induced ROS that leads to hyper activation of AKT, inhibition of pro-apoptotic protein BAD and caspase 9, and activation of mitogenic transcription factors such as ASK1 and GSK3 has also been reported [43]. In contrast, an increased ROS level with resulting oxidative stress is known to induce cell death by apoptosis induction mediated by Fas ligand [44], [45] and/or p53 [44]. Dose-dependent dual function of ROS, that is, low dose-induced growth and high dose-induced cell apoptosis in acute exposure as observed in this study is consistent with these previous reports. In addition, the adaptation of breast cancer cells to chronic exposure to high dose of ROS, that was cytotoxic in acute study, suggest that persistent exposure to even higher dose of ROS eventually confer resistance to cell death. Mechanistically, this adaptive response was associated with reversal of ROS-mediated gene regulation that induces apoptosis in acute exposure. For example, pro survival genes *Bcl2, Survivin* and *Cyclin D1* genes were down regulated in acute exposure, whereas, there was no significant change in the expression of these genes in cells chronically exposed to H_2_O_2_ as compared to untreated control.

Metastasis is one of the characteristic features of aggressive form of malignant neoplasm and also the leading cause of mortality in breast cancer patients. In addition to the increased growth and survival, we also analysed the expression of genes involved in metastatic potential of MCF-7 breast cancer cells. The increased expression of pro-metastatic genes such as *VEGF, CD44* and *WNT1*; decreased expression of anti-metastasis gene *E-cadherin* following chronic exposure to oxidative stress suggests that ROS not only increases growth and survival, but can also increase metastatic potential of breast cancer cells. Multiple studies have shown higher levels of ROS in metastatic tumor cells than normal and primary malignant cells [46]–[48] and significant reduction in metastasis potential with increased levels of antioxidant compounds and ROS metabolizing enzymes [49]. Increased production of ROS due to mutations in mitochondrial DNA has been shown to contribute in metastatic potential of human breast cancer cells [50]. Similarly, angiogenesis is a vital factor for tumor growth and metastasis. *VEGF* is one of the major angiogenesis factors and is known to stimulate angiogenesis by inducing endothelial cell proliferation and migration [48], [51]. These previous reports and up-regulation of *VEGF* in H_2_O_2_ exposed cells as observed in this study further supports the role of ROS in metastatic potential of cancer cells. ROS is believed to modulate molecular cross talk between cell adhesion proteins like cadherins and integrins and is one of the important process for cell to cell adhesion [52]. Our data also revealed decreased expression of *E-cadherin* in H_2_O_2_ exposed cells. *E-cadherin* is an epithelial cell adhesion molecule which is known to play key role in initial stage of metastasis [53] and has been associated with poorly invasive and poorly differentiated breast cancer phenotype [54]. Down regulation of *E- cadherin* by estrogen-induced ROS has also been reported [55]. The result of this study showing decreased expression of *E- cadherin* in H_2_O_2_ exposed cells is consistent with these previous reports and further supports the role of increased ROS in mediating the cancer cell metastasis by decreasing the levels of E*- cadherin*. *WNTs* are another class of protein that regulate initiation, progression, differentiation and metastasis of cancer cells [56]. Up regulation of *WNT1* following chronic exposure to high dose H_2_O_2_ further strengthens our findings. Sub-chronic (two to four days) exposure to oxidative stress has been reported to increase invasive potential in mouse mammary gland epithelial cells with morphological alterations suggesting epithelial to mesenchymal transition along with redistribution of *E-cadherin* and up-regulation of *MMPs* [57]. Therefore our present findings of ROS-induced changes in marker genes for cancer cell adhesion and metastasis along with these previous reports suggest the role of ROS in cancer cell metastasis. Additionally, this study has provided the evidence for direct involvement of ROS in cancer cell metastasis by regulating the genes involved in metastatic pathway.

Interestingly our data suggest that higher dose of ROS causes cell toxicity in acute exposure whereas the adaptability of those cells that survives acute toxicity of ROS increases due to persistent exposure to ROS. Previous studies have shown that increased adaptability of cancer cells to chemotherapy is because of selective growth advantage of cancer stem cells but not the other cancer cells [3]. Several reports suggest the presence of cancer stem cell (CSC) in breast cancer cell population and were considered to be responsible for progression [58], metastasis [59], and resistance of cancer cells to chemotherapy [60]. Exposure of genetically unstable cancer cells to selective tumor micro-environment pressures including increased ROS has been proposed for cancer stem cell formation through epithelial to mesenchymal transition by genetic and epigenetic changes [61]. Increased expression of oxidative stress-responsive genes has been reported in breast cancer stem cells and has also been implicated partly for the ability of cancer stem cells to survive and resist anticancer therapy [62], [63]. Expression of *CD44* is known to be higher in cancer stem cells than the remaining cancer cells or parental cancer cells [3], [43]. For example, increased expression of *CD44* has been reported in tumorsphere cultures of MCF-7 cells [64]. Up regulation of *CD44* in association with elevated levels of ROS was also reported in aggressive phenotype of nasopharyngeal carcinoma [65]. Increased expression of *CD44* with down regulation of *E-cadherin* and increased number of soft agar colony formation in MCF-7 cells chronically exposed to H_2_O_2_ in our study is consistent with previous reports. These reports further strengthens role of oxidative stress in CSC development. These reports and the present finding of increased expression of cancer stem cell markers such as *CD44*, and decreased expression of *E-cadherin* (suggesting epithelial to mesenchymal transition) in H_2_O_2_ exposed cells suggest a potential mechanism for increased adaptability of cancer cells to ROS toxicity by selection of cancer stem cells. Further study is needed to understand how these cancer stem cells tolerate ROS induced toxicity and to identify epigenetic changes, if any, that provides them advantage in tolerating ROS.

In addition, the result of this study showing increased survival and tumorigenicity of MCF-7 cells due to chronic oxidative stress may also have implication in understanding the acquired resistance to chemotherapeutic drugs in breast cancer cells due to longer treatment. Certain chemotherapeutic agents produce ROS and therefore cause oxidative stress in cancer cells [66], [67]. Some of the breast cancer chemotherapeutic drugs shown to induce oxidative stress include Tamoxifen, Doxorubicin, Mitomycin C, Etoposide and Cisplatin [31]. Recent studies suggest that cellular adaptation to oxidative stress through activation of redox sensitive transcription factors may be a mechanism for both intrinsic and acquired resistance to multiple anticancer agents. Additionally, resistance of cancer cells to these drugs was shown to be correlated with increased antioxidant capacity [68], [69]. It was reported that these chemotherapeutic drugs-induced oxidative stress can generate cell senescence with emergence of a cell population with stem cell activity to evade chemotherapy [70]. In addition, recent reports show that anticancer activity of Paclitaxel against breast cancer cells is mediated by increased ROS production through NADPH oxidases (NOX) activation [71]. Hence, findings of this study showing increased cell survival and adaptation to chronic oxidative stress of H_2_O_2_ exposed cells may also explain a mechanism for resistance of breast cancer cells to chemotherapeutics which induce cytotoxicity mainly by elevating ROS levels in cancer cells. In summary the finding of this study provides evidence for direct role of ROS in inducing growth and metastatic potential in breast cancer cells. Additionally, this study suggest that resistance to ROS-inducing chemotherapeutic drugs in breast cancer could at least in part be due to selective growth advantage of cancer stem cells in oxidative stress environment. These findings will open a new avenue for designing or developing a new strategy for breast cancer treatment by targeting redox signalling through antioxidants.
