# Correction: Chronic Oxidative Stress Increases Growth and Tumorigenic Potential of MCF-7 Breast Cancer Cells

**DOI:** 10.1371/journal.pone.0093799

**Published:** 2014-04-01

**Authors:** The PLOS ONE Staff

There is an error in Figure 2. Please view the correct version here:

**Figure 2:**
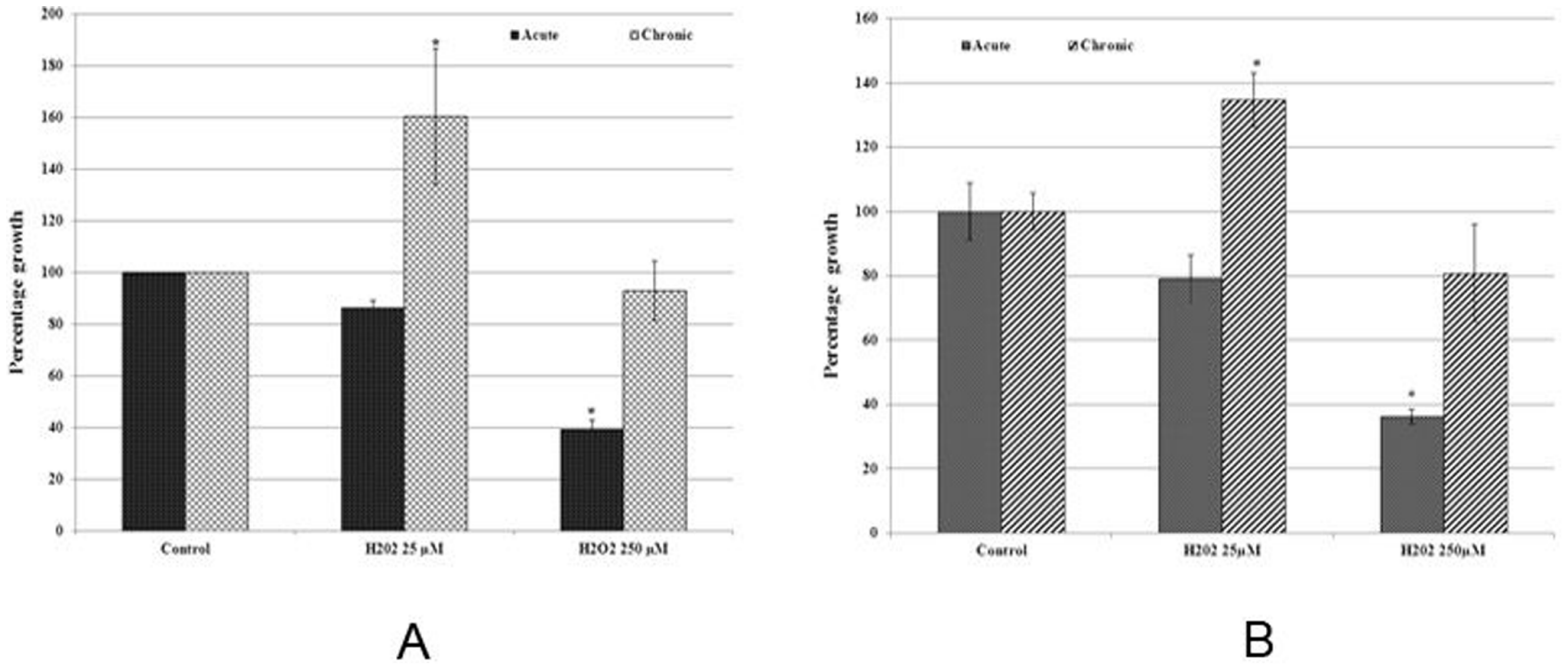
Bar graph representation of cell growth data from cell count analysis (Figure 2A), and MTT assay (Figure 2B) of MCF-7 cells with acute and chronic exposure to H_2_O_2_. Values for cell count and MTT assay were converted into percentage of control (control  =  100%). The error bars represent the standard error of the mean (±SEM). Statistically significant (p<0.05) changes are indicated by symbol *.
